# Pharmacological management of perioperative bleeding in cardiac surgery

**DOI:** 10.21542/gcsp.2017.12

**Published:** 2017-06-30

**Authors:** Hossam Walley, Magdi Yacoub, Hesham Saad

**Affiliations:** Aswan Heart Centre, Aswan, Egypt

## Background

In spite of the growing discoveries in cardiac surgery, perioperative bleeding remains one of the most common causes of morbidity and mortality.^1^ Around 50-60% of cardiac surgery patients receive blood transfusions,^2,3^ which are harmful and strongly associated with increased morbidity, mortality, hospital stay, and hospital cost.^4–7^ Patients taken back to the OR for re-exploration and control of bleeding after cardiac surgery, have a four-fold increase in the incidence of sternal infection and mortality.^8^

These facts highlight the pressing need of developing further strategies to deal with the problem. Pharmacological management of perioperative bleeding has evolved rapidly over the last few years.^9^

Antifibrinolytics remain a cornerstone of the pharmacologic part of the multimodal blood conservation program.^9^ The use of antifibrinolytics (aprotinin, tranexamic acid and ε-aminocaproic acid) in the last two decades is associated with many myths and facts. Choosing which antifibrinolytic agent, to give to which patient, at what dose, remains the ultimate goal for cardiac anesthesiologists.

The recent trial by Paul S. Myles et al. published in the New England Journal of Medicine,^10^ represents a welcome addition to the literature. In this trial, for patients undergoing coronary-artery surgery, tranexamic acid was associated with a lower risk of bleeding than a placebo, without a higher risk of death or thromboembolic complications within 30 days of surgery. However, tranexamic acid was associated with a higher risk of postoperative seizures.^10^

We review this particular trial, and the rapidly evolving field of pharmacological management of perioperative bleeding - both in terms of efficacy limitation, and other future discoveries.

## Fibrinolysis and cardiac surgery

Surgery results in tissue damage and blood loss, and as a result the homeostatic mechanisms are activated. Hemostasis depends on the balance of four processes: anticoagulants and fibrinolysis - which promote bleeding, and procoagulant and anti-fibrinolysis - which promote coagulation. Imbalance between the four processes results in either excessive bleeding or thrombosis, or both.^11^ At the surgical site, the fibrinolysis activity has been shown to increase 4-8 fold. The degree of fibrinolysis activation depends on the amount of tissue injury and the type of surgery.^12^

**Figure 1.**
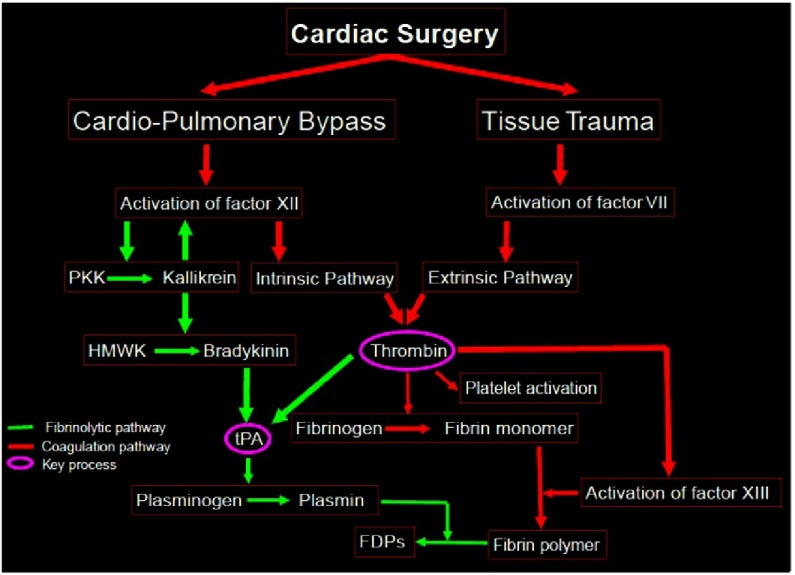
Flow diagram depicting activation of coagulation and fibrinolysis during cardiac surgery^11^.

Cardiac surgery, with its combination of massive tissue injury and use of cardiopulmonary bypass, disturbs the harmony of the hemostasis processes. The flow diagram below depicts the activation of the coagulation and fibrinolysis during cardiac surgery [Figure 1].^11^

Plasminogen is activated by tissue plasminogen activator (t-PA) into plasmin, and plasmin furthers degrades fibrin into fibrin degradation products.^12^

Studies have shown a five-fold increase in active tissue plasminogen activator level during cardiopulmonary bypass, tissue plasminogen activator progressively increases during cardiopulmonary bypass and remains elevated for up to two hours afterwards.^13^ This increased fibrinolysis consumes fibrinogen, leaving very little available for coagulation.

## Antifibrinolytics and cardiac surgery

As mentioned above, the increased level of tissue plasminogen activator and plasmin during cardiopulmonary bypass has resulted in increased use of antifibrinolytic agents in cardiac surgery [Table 1].^14^

**Table 1:** Antifibrinolytics agents: drug description, doses, and mechanism of action.^14^

Drugs	Composition	Mechanism of action	Elimination	Pharmacodynamics	Suggested dosing in adults	Approval
Aprotinin	Protein, isolated from bovine lung tissue	Protease inhibitor; reversibly complexes with the active sites of plasmin, kallikrein, and trypsin; inhibition of fibrinolysis, activated factor XIIa, thrombin-induced platelet activation, and inflammatory response	Predominantly proteolysis, <10% renal	Initial plasma half-life 150 min and terminal half-life 10 h	1. “Full dose”: 2 × 10^6^ KIU bolus patient, 2 × 10^6^ KIU bolus CPB, continuous infusion of 5 × 10^5^ KIU. 2. “Half dose”: 1 × 10^6^ KIU bolus patient, 1 × 10^6^ KIU bolus CPB, continuous infusion of 2.5 × 10^5^ KIU	Suspended since 2008; suspension lifted in Canada in 2011 and Europe in 2012; In the United States still suspended
Tranexamic acid	Synthetic lysine analog	Antifibrinolytic; competitive inhibition of the activation of plasminogen to plasmin	Renal	Plasma half-life 3 h	1. “High dose”: 30 mg/kg bolus patient, 2 mg/kg CPB, and continuous infusion of 16 mg/kg; 2. “Low dose”: 10 mg/kg bolus patient, 1-2 mg CPB, and continuous infusion of 1 mg/kg	United States, Canada, Europe
ε-Aminocaproic acid	Synthetic lysine analog	Antifibrinolytic; competitive inhibition of the activation of plasminogen to plasmin	Renal	Plasma half-life 2 h	100 mg/kg bolus patient, 5 mg/kg CPB, and continuous infusion of 30 mg/kg	United States, Canada

**Notes.**

CPB cardiopulmonary bypass KIU Kallikrein International Unit

Aprotinin (a non-specific serine protease inhibitor) inhibits plasmin in a high dose and is the only agent shown to reduce the need for re-exploration.^15^ However, marketing of the drug was suspended in November 2007 after preliminary results of the BART trial, which showed an increasing mortality trend relative to the lysine analogues.

Lysine analogues (ε-aminocaproic acid and tranexamic acid) were frequently used to competitively inhibit the fibrin-binding site on plasminogen, thus reducing the rate of fibrinolysis. Compared to placebo, ε-aminocaproic acid and tranexamic acid reduce the total blood loss and decrease the number of patients requiring blood transfusion.^16^ The Society of Cardiothoracic Surgeons and the Society of Cardiovascular Anesthesiologists updated the 2011 guidelines on blood conservation, which recommend the use of lysine analogues (class 1A evidence).^9^

## Cracking the aprotinin dilemma

Aprotinin, in high doses, is the only agent shown to reduce re-exploration after cardiac surgery.^15^ In 2006, the question of aprotinin use in cardiac surgery was raised after the publication of two observational studies. The first, conducted by Mangano et al.,^17^ showed that aprotinin is associated with increased risk of cardiovascular events, cerebrovascular events and renal dysfunction. The second study was conducted by Karkouti et al.,^18^ and showed an increased risk of renal toxicity with the use of aprotinin [Table 2].^14^

**Table 2:** Summary of studies that assessed the safety of Aprotinin.^14^

Authors	Year	Design	Aprotinin (n)	Control (n)	Death	Kidney dysfunction	Kidney failure
Karkouti et al.^2^	2006	Case–control propensity	449	TXA: 449	NS	↑	NS
Mangano et al.^3^	2006	Observational propensity	1,295	TXA: 822; EACA: 823	↑	↑	↑
Schneeweiss et al.^4^	2008	Retrospective	33,517	EACA: 44,682	↑	NS	NS
Shaw et al.^5^	2008	Retrospective	1,343	EACA: 6,776	↑	↑	↑
Fergusson et al.^8^	2008	RCT	781	TXA: 770; EACA: 780	↑^*^	NS	NS
Karkouti et al.^6^	2010	Retrospective propensity	1,017	TXA: 1,544	NS	NS	↑^†^
Walkden et al.^7^	2013	Case–control propensity	1,754	TXA: 1,754	NS	↓^‡^	↓^‡^

**Notes.**

↑ is increased with aprotinin; ↓ is increased in control.

* *P* = 0.05 for aprotinin vs. TXA and *P* = 0.06 for EACA.

† Aprotinin significantly increased the incidence of acute kidney injury in low-risk and intermediate-risk patients (*P* = 0.006), and no difference observed in high-risk patients (*P* = 0.8).

‡ Difference observed in the whole population, but no difference when considering only high-risk patients.

EACA ε-Aminocaproic acid NS not statistically significant RCT randomized controlled trial TXA tranexamic acid

However, these two studies were observational, non-randomized, and both compared aprotinin to products that were not FDA-approved at that time.

In 2007, Bayer released the results of a self-commissioned observational study showing an increase in kidney damage, congestive heart failure, stroke, and mortality.^19^ At the same time, the BART study (the blood conservation using antifibrinolytics in a randomized trial) showed increased mortality with aprotinin compared to lysine analogues.^20^ In November 2007, Bayer temporarily suspended marketing of the drug at the request of Health Canada. By May 2008, Bayer had removed the remaining stocks and withdrawn aprotinin from the worldwide market.

Proponents of aprotinin refused to let it’s withdrawal from the market be the end of the story. They felt there much needless suffering by patients by not being able to use aprotinin. Hence, questions were raised about the research methods and data analysis of the BART trial.^21–23^

In December 2008, Health Canada set up an expert advisory panel to discuss the benefit/risk issues of aprotinin and the panelists were asked very specific questions.^24^

The panel found some of the hidden mystery behind the BART trial

1- The primary outcome in the BART trial was risk of massive bleeding, not mortality.
2- The trial was not sufficiently powered to detect the difference in mortality.
3- Even though the BART trial was proposed for high-risk patients, the panel found that the patients belonged to the moderate risk group. High-risk patients were either not enrolled or excluded from the trial.
4- The data of 137 patients excluded after randomization was requested and provided by the researchers. The aprotinin group in this subgroup showed lower mortality and no satisfactory explanation was provided.
5- Combining data from both the included and the excluded patients showed that mortality in the aprotinin group was not statistically significantly higher and could have occurred by chance.

The panel concluded that risk/benefit ratio for aprotinin was still favorable as the trials for its initial approval were still relevant. However, further studies were needed to define the risk/benefit of aprotinin. The panel did not find any advantage of aprotinin in low-risk patients, with an expected blood transfusion of one to three units. In these situations, blood transfusion would be safer than aprotinin.^24^

In September 2011, Health Canada sent a letter to all heath professionals informing them of the lifting of the temporary suspension of aprotinin in Canada. Aprotinin has been authorized to be used only for isolated CABG surgery in Canada, and only after careful consideration of the potential risks and benefits.^24^

## Are tranexamic acid and ε-aminocaproic acid adequate substitute for aprotinin?

### Analogues of the amino acid lysine

Tranexamic acid and ε-aminocaproic acid are synthetic antifibrinolytic amino acids that competitively block the lysine-binding site of both plasminogen and plasmin, therefore inhibiting each enzyme action. Plasmin can no longer bind to fibrin and can no longer degrade fibrin, and thus bleeding is reduced.^14^

Tranexamic acid and ε-aminocaproic acid have a small molecular weight and half life of about two to three hours. On a molar basis, TA is at least seven times more potent than EACA.^14^

TA is more effective relatively to EACA in reducing the need for blood transfusion in cardiac surgery. TA saved an average of 300 ml of blood per patient during cardiac surgery, with relative risk reduction of 32% in receiving blood transfusion. EACA saved an average of about 200 ml of blood during cardiac surgery, with 30% relative risk reduction in blood transfusion.^16^

As mentioned by Beverly Hunt in 2014 “*There has been an explosion of interest in the ability of tranexamic acid to reduce morbidity and mortality in surgical and traumatic bleeding. Tranexamic acid has been shown to reduce mortality due to traumatic bleeding by a third, without apparent safety issues. It is now clearly established that intravenous tranexamic acid reduces blood loss in patients with surgical bleeding and the need for transfusion”.*^25^

The Myles et al. trial,^10^ with 2-by-2 factorial design, randomly assigned patients who were scheduled to undergo coronary-artery surgery and were at risk for perioperative complications, to receive aspirin or placebo and tranexamic acid or placebo. The primary outcome was a composite of death and thrombotic complications (non-fatal myocardial infarction, stroke, pulmonary embolism, renal failure, or bowel infarction) within 30 days of surgery.

No evidence was found that the use of tranexamic acid resulted in a higher risk of death or thrombotic complications than with placebo. They also found that the tranexamic acid group had a lower risk of blood loss, blood transfusion, and reoperation, but a higher risk of postoperative seizures, than the placebo group.^10^

The most interesting question based on previous data is, “Are the analogues of the amino acid lysine adequate substitutes for aprotinin?”

Sander et al.,^26^ compared high dose TA to aprotinin and found that the TA group was associated with an increased rate of re-exploration, chest tube drainage and postoperative seizures, while the aprotinin group was associated with increased rate of late ischemic stroke and neurological disorders.

**Figure 2.**
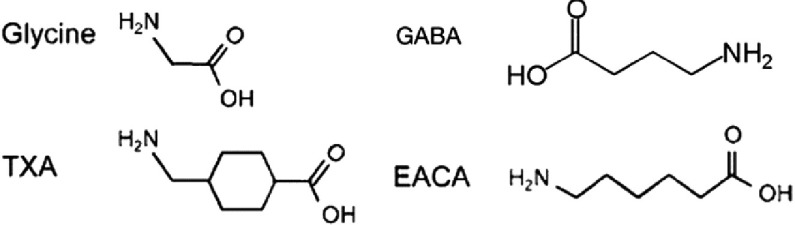
Chemical structure of antifibrinolytics, glycine, and GABA^14^.

Postoperative seizures reported after the use of TA was an alarming sign about the use of TA in cardiac surgery. Furtmuller et al.,^27^ explain that the seizures associated with the use of lysine analogues could be due to γ-aminobutyric acid (A) [GABA] receptor antagonism with possible involvement of other receptors. Figure 2 showing the similarities in the chemical structures of tranexamic acid, GABA, and glycine.^14^ Iplikciglu and Berkman suspect that cerebral ischemia caused by either vasospasm or thrombosis may be the cause of TA-induced seizures.^28^ Table 3 summarizes studies assessing the seizures and mortality associated with tranexamic acid.^14^

**Table 3:** Tranexamic acid: seizures and mortality.^14^

	TXA Dose	Patients (n)	Seizures			Mortality		
			Control (%)	TXA (%)	*P* Value	Control (%)	TXA (%)	*P* Value
Sander et al.^26^	50 mg/kg bolus; 50 mg/kg CPB	Total = 893; TXA = 336; Aprot = 557	0.9	2.7	0.05	6.9	8.6	0.34
		Open: Heart = 320, TXA = 105, Aprot = 215	1.9	6.7	0.04	7.5	16.2	0.02
Koster et al.^27^	1 g bolus/0.5 g CPB/ infusion 0.2 g/h	Total: N = 4,883, TXA = 1,029, Aprot = 3,854	1.2	1.8	0.32	1.1	1.5	0.446
		Open: Heart = 2,779, TXA = 636, Aprot = 2,143	1.3	3.0	0.04	1.7	5.7	<0.001
Makhija et al.^28^	10 mg/kg bolus/infusion 1 mg kg^−1^ h^−1^	TXA = 31, EACA = 30	3.3	10	0.19	0.0	6.4	0.49
Martin et al.^29^	2 g bolus/2 g CPB/ infusion 0.5 g/h	TXA = 275, EACA = 329	3.3	7.6	0.019	5.0	4.7	0.899
Martin et al.^30^	50 mg/kg boluses before and after CPB; 100 mg/100 ml CPB prime	TXA = 114, EACA = 120	0.8	3.5	0.203	3.3	2.6	0.999

**Notes.**

Aprot aprotinin CPB cardiopulmonary bypass EACA ε-aminocaproic acid TXA tranexamic acid

## What is the proper dose of tranexamic acid in cardiac surgery?

Despite a wide variety of dosing regimens being examined,^11^ this controversial question has never been properly answered.

Armelin et al.,^29^ compared the low and high doses of TA and found no difference in blood loss or transfusion requirements. The plasma concentration of TA that is required to inhibit fibrinolysis *in vitro* is 10 μg/ml.^30^ The dose of TA that is needed to maintain plasma concentration of above 20 μg/ml has been calculated as: Loading dose: 5.4 mg/kg, CPB prime dose: 50 mg for 2.5 L circuit, and rate of infusion: 5 mg/kg/h, with adjustment to the loading and prime dose in renal insufficiency.^31^

Dowd et al.,^32^ studied the pharmacokinetics of TA during cardiopulmonary bypass and they found that a TA concentration of 127 µmol is sufficient to provide >90% inhibition of the tissue activators of fibrinolysis and is the minimum therapeutic plasma concentration.

In the CRASH-2 trial,^33^ even a very low dose (bolus of 1 g followed by infusion of 1 g over eight hours) was effective in reducing the all-cause mortality. However, another randomized trial by Bokesch et al.,^34^ reported greater efficacy, fewer seizures, and lower mortality, with the same higher dosage as used in the BART trial.

In the Myles et al. trial,^10^ they used a dose of 100 mg/kg at the beginning of the trial, but later reduced this dose because of the growing number of reports of seizures associated with tranexamic acid that were believed to be dose-related. The smaller dose (50 mg/kg) did not reduce the risk of seizures.

## Outlook on genomics and future

Despite an improved understanding of the hemostasis process during cardiopulmonary bypass, it is very difficult to predict which patient is at a higher risk for bleeding or thrombosis. Defects in coagulation proteins, like plasminogen activator inhibitor-1, factor VII, fibrinogen, and t-PA, have a strong heritability profile.^35^ Welsby et al.,^36^ identified seven genetic polymorphisms associated with bleeding after cardiac surgery and concluded that although genetic factors appear primarily independent, combining genetic and clinical factors doubles the ability to predict bleeding after cardiac surgery. Accounting for genotype may be necessary when stratifying risk of bleeding after cardiac surgery.

## What we have learned?

Perioperative bleeding in cardiac surgery is not just a surgical problem, but a combination of surgical, medical, and hereditary problems. Facing perioperative bleeding in cardiac surgery is not a death sentence, but an alert to the surgeon, anesthesiologist, and intensivist to corroborate for its management. The prediction of the high-risk patient along with the use of antifibrinolytics may catch the problem but with cost. Choosing which antifibrinolytics to which patient at what dose should be applied for every patient individually according to the risk/benefit ratio regardless the cost-effectiveness.
